# A broadband detector based on series YBCO grain boundary Josephson junctions

**DOI:** 10.3762/bjnano.13.27

**Published:** 2022-03-28

**Authors:** Egor I Glushkov, Alexander V Chiginev, Leonid S Kuzmin, Leonid S Revin

**Affiliations:** 1 Institute for Physics of Microstructures of RAS, GSP-105, Nizhny Novgorod, 603950, Russia; 2 Nizhny Novgorod State Technical University n.a. R. E. Alekseev, GSP-41, Nizhny Novgorod, 603950, Russia; 3 Chalmers University of Technology, SE-41296 Gothenburg, Sweden

**Keywords:** array, electromagnetic modeling, log-periodic antenna, RCSJ model, series Josephson junctions, YBaCuO Josephson junction

## Abstract

Modeling of a broadband receiving system based on a meander series of Josephson YBaCuO grain boundary junctions integrated into a log-periodic antenna was carried out. The electromagnetic properties of the system, namely amplitude–frequency characteristic, beam pattern, and fraction of the absorbed power in each Josephson junction were investigated. Based on the obtained results, a numerical simulation of one-dimensional arrays was carried out. The dc characteristics of the detector were calculated, that is, current–voltage characteristic, responsivity, noise, and noise-equivalent power (NEP) for a 250 GHz external signal. The optimal number of junctions to obtain the minimum NEP was found. The use of a series of junctions allows one to increase the responsivity by a factor of 2.5, the NEP value by a factor of 1.5, and the power dynamic range by a factor of 5. For typical YBaCuO Josephson junctions fabricated on a ZrYO bicrystal substrate by magnetron deposition, the following parameters were obtained at a temperature of 77 K: responsivity = 9 kV/W; NEP = 3·10^−13^ W/Hz^(1/2)^; power dynamic range = 1·10^6^.

## Introduction

High-temperature superconducting (HTSC) Josephson junctions (JJs) have great potential as promising materials for creating high-frequency devices, such as microwave generators [1–2], sensitive detectors or mixers [3–4], and voltage standards [5–7]. Recent impressive developments in this area make this possible [8–10].

The advantage of HTSCs lies not only in the ability to operate at liquid-nitrogen temperature, but also in a wide temperature range. For instance, an YBaCuO Josephson detector was applied to detect a small signal from a BiSrCaCuO mesa structure at an operating temperature of 25 K [11]. A HTSC JJ has also analyzed pulsed terahertz radiation from quantum cascade lasers located in the same cryogenic environment at 50 K [12]. Another important advantage of HTSCs is a wide frequency range from tens of gigahertz to several terahertz [13–14], which exceeds the gap limitation of low-*T*_c_ detectors. So, in [12] the spectrum of a quantum cascade laser emission has been recovered by an YBaCuO detector at a central frequency around 2.2 THz.

However, for some applications the performance of the HTSC devices is limited by the fairly low impedance of the JJ. It leads to moderate conversion efficiency in Josephson mixers [15–16] and reduced absorbed power in JJ detectors [17]. One possible way to solve this problem is to replace a single JJ by a chain or an array of JJs combined with planar coupling structures. The task of developing and calculating such systems is quite complex and faced with obscures such as unresolved Shapiro steps [18–19] and parasitic resonances [3].

To effectively match JJs with open space and the incoming external signal, dipole antennas for each junction can be used [20], but the development of a broadband detector requires the use of spiral [4], log-periodic [3], or other types of broadband antennas. In this case JJs are integrated into the single receiving system. This is the case considered in our article.

The aim of this work is the electromagnetic analysis and numerical simulation of the broadband detector based on a series of HTSC bicrystal Josephson junctions to maximize response, power dynamic range, and noise-equivalent power.

## Electromagnetic Simulation

**Figure 1:**
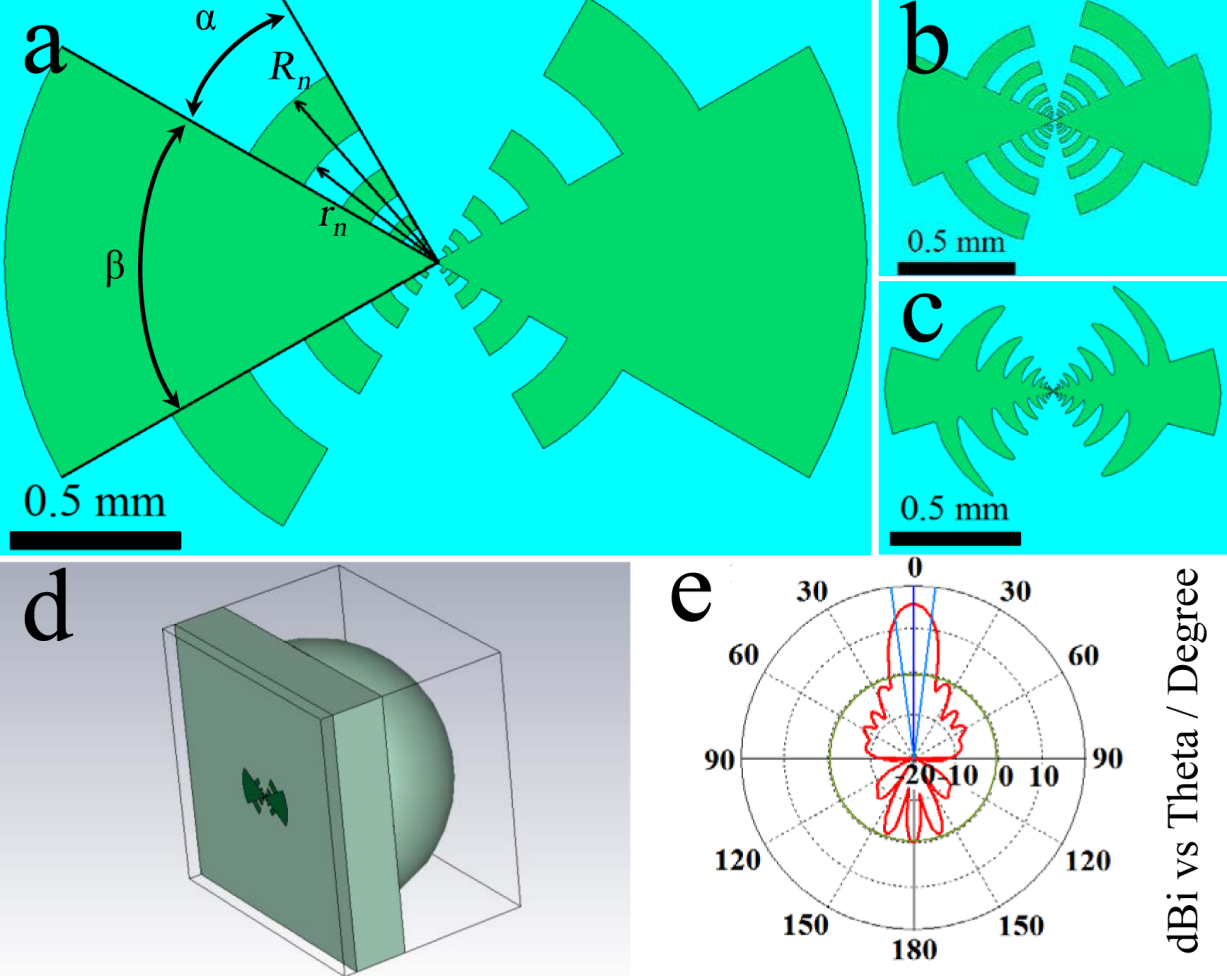
(a–c) Three log-periodic antennas of various geometries; (d) system with a lens (d) and (e) beam pattern at 250 GHz.

Figure 1a–c shows options for the geometry of a log-periodic antenna. Its main advantage is a wide frequency band. The basic formulas and parameters that define this type of antenna are the angles α and β, determining the length of the teeth, the number of teeth *n* and their radii [21–22]:


[1] $$R_{n}=\tau^{\left(n-\frac{1}{2}\right)}r_{1},\;r_{n+1}=\tau^{n}r_{1}.$$

For the geometry in Figure 1c with a sinuous modulation of the antenna edges, additional expressions were used [23].

The tool used for the simulation was time domain solver of CST Microwave Studio. Two methods for calculating the receiving characteristics of the antenna were used. In the first method, microwave (MW) currents and voltages excited in each receiving element (port) were calculated for the case that a plane wave was incident on the structure through the lens. In the second method, the full matrix of the antenna input impedance *Z* was calculated for all ports in the emission regime. The first calculation method is well applicable for low frequencies, while for high frequencies the duration of calculations increases significantly with a decrease in the step of the computation grid. The second method works well for a small number of ports; with an increase in the number of receiving elements, the computation time for the complete *Z*-matrix increases. Both methods give similar results with acceptable accuracy.

**Figure 2:**
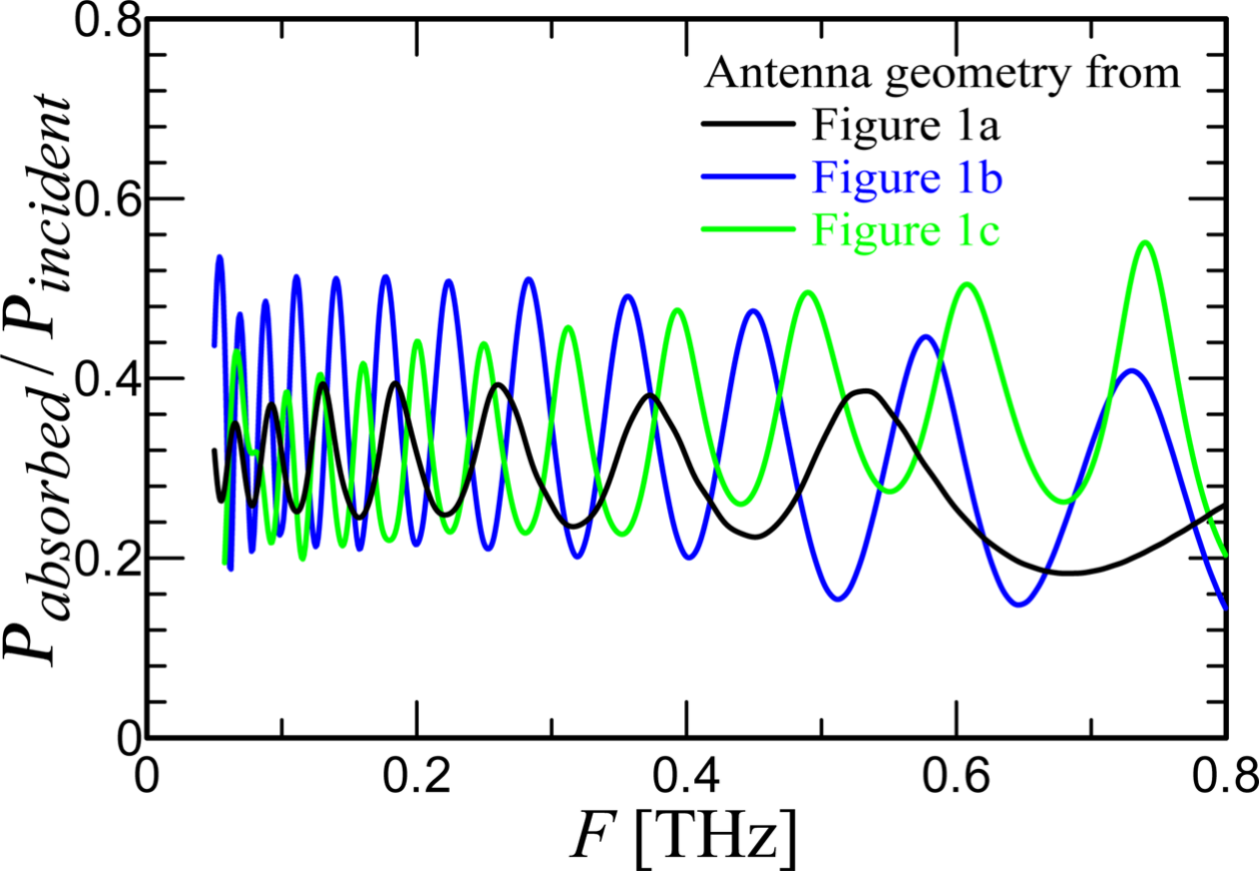
Normalized level of absorbed power in the port for the three antenna geometries in Figure 1.

Figure 1 shows a complete modeling system with lens (Figure 1d) and calculated beam pattern at 250 GHz (Figure 1e). The obtained values of 15.7 dBi main lobe magnitude with 15° angular width demonstrate good receiving characteristics. A port with an impedance of 5 Ω was used as an element simulating the Josephson junction. It is known [17,24] that the impedance of the HTSC JJ is low, which makes the development of HTSC detectors and mixers difficult in comparison with low-temperature superconductor Josephson junction analogues. Figure 2 shows the dependence of the received power *P*_absorbed_/*P*_incident_ as function of the frequency for the three different antenna geometries from Figure 1. It can be seen that this value does not reach 1 due to the mismatch of the antenna impedance (about 50 Ω) and the port impedance. The differences in the results for the three antenna geometries are explained by the influence of the antenna parameters on the S-parameters. τ affects the frequency distance between resonances, and the length of the teeth (combination of α and β) affects the depth of the resonances. For the antennas in Figure 1b,c τ is smaller than for the antenna in Figure 1a. Therefore, more resonances are within the range of 50 to 800 GHz. In addition, the teeth for these two designs are longer, so the maxima of the absorbed power at the resonant frequencies are greater. At the same time, the power drops at non-resonant frequencies increase, making the amplitude–frequency characteristic (AFC) less smooth. Since the purpose of the current work was to develop a broadband system, for further consideration we chose an antenna with the most uniform AFC, sacrificing the maximum absorbed power in resonances. This is the antenna in Figure 1a with the parameters α = 30°, β = 60°, *r*_1_ = 28 μm, τ = 2, and *n* = 5.

**Figure 3:**
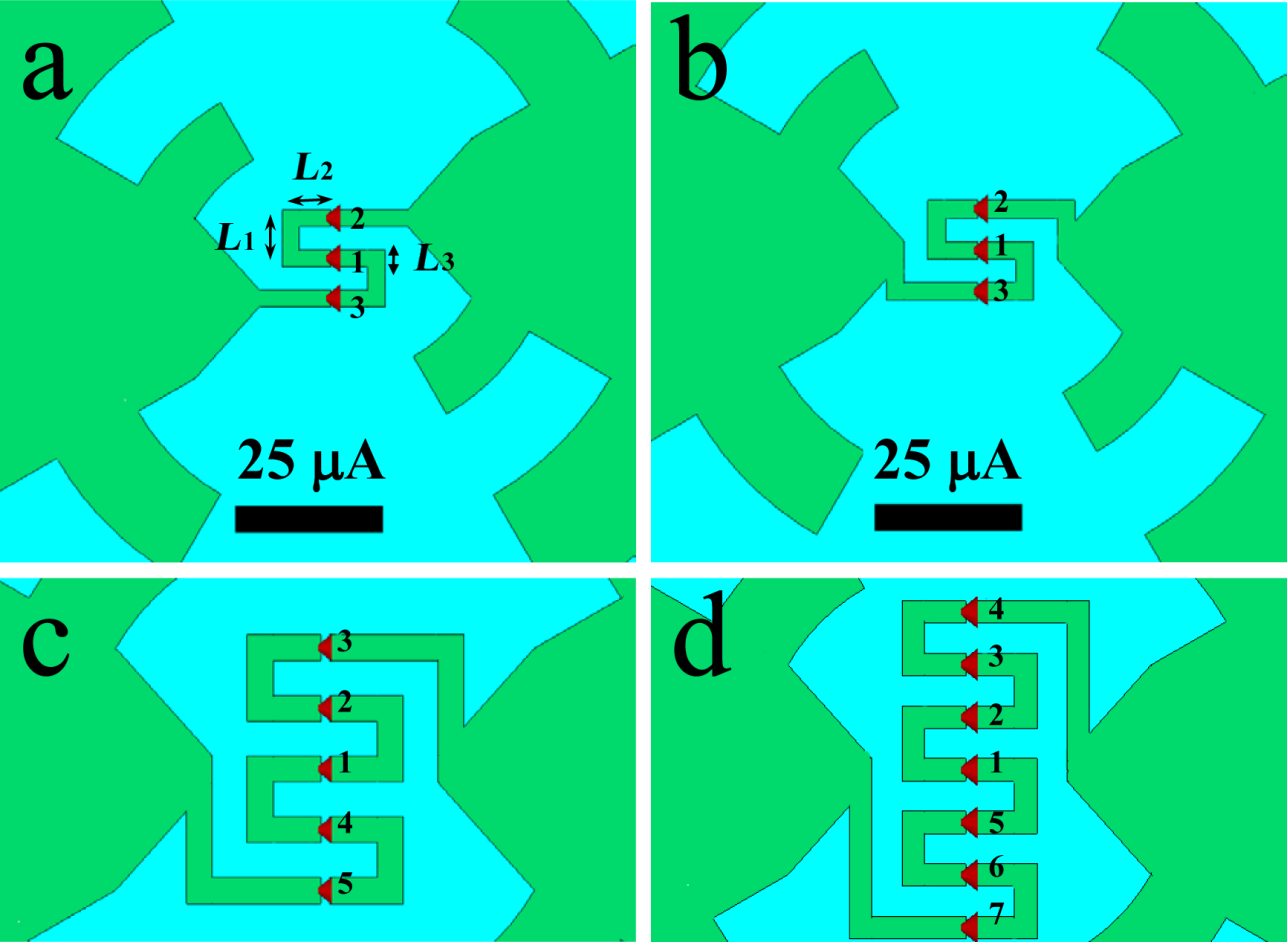
Geometry of log-periodic antennas with a meander series of Josephson YBaCuO grain boundary junctions. (a) Displaced geometry with three ports; (b) unchanged antenna with long meander and three ports; (c, d) antennas with five and seven ports, respectively.

To improve the match between receiving system and antenna, designs with series of up to eleven Josephson junctions were considered. In this case, there are two possible ways to implement the geometry (Figure 3a,b). The fabrication of bicrystal junctions requires the use of a meander-type microstrip since, for the formation of a weak link, it is necessary for the superconducting film to cross the grain boundary. In the first case implemented in [3], the length of the meander is minimal (Figure 3a). To minimize this length, the two parts of the antenna are displaced vertically relative to each other. The disadvantage of this approach is that with an increase in the number of ports and, accordingly, an increase in the meander length, the vertical displacement of the antenna parts becomes noticeable, which is detrimental to its receiving properties. In the case of the geometry in Figure 3b, the antenna itself remains unchanged, and the meander becomes longer compared with the first case. This design will not violate the concept of a log-periodic antenna, but due to the proximity of the meander microstripes to the tooth with *n* = 1, it may lead to distortion of the AFC at high frequencies. Figure 3c,d shows antennas with five and seven ports, respectively. Due to the central symmetry of such a geometry, the receiving properties of the ports above and below the central one will be the same, thus, ports 2 and 3 in a three-port antenna will be equally excited. For an antenna with five ports, *S*_22_ = *S*_44_, *S*_33_ = *S*_55_ and so on.

**Figure 4:**
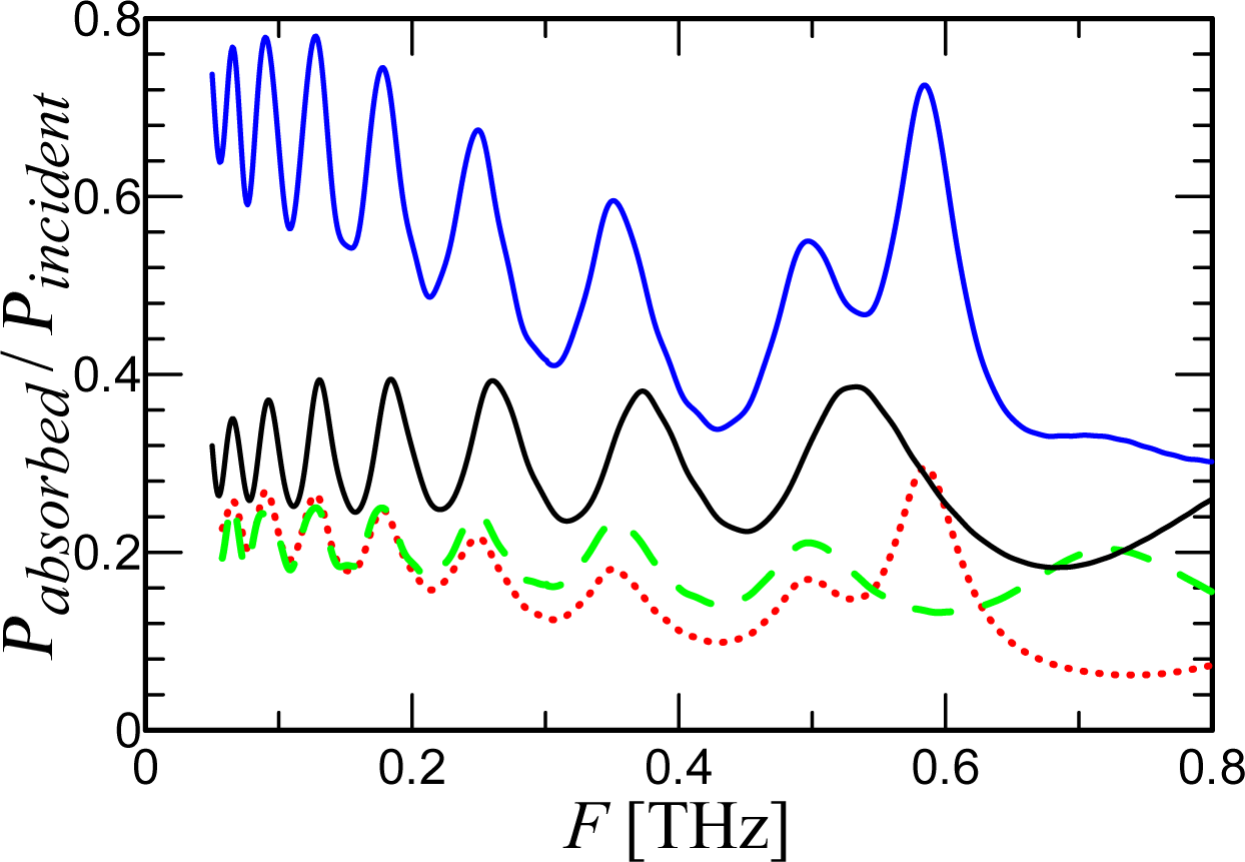
Integral absorbed power for two different cases. Black curve: a single port in an antenna; blue curve: three ports in series. Green dashed line: *P*_absorbed_ in the central port of the three-port configuration. Red dotted line: *P*_absorbed_ in port 2 and port 3.

Figure 4 shows a comparison of the simulation results for one receiving element and three receiving elements in series. It can be seen that the integral power absorbed in three ports (blue curve) is greater than the power absorbed in one port (black curve). For low frequencies, the power received by three ports with an impedance of 5 Ω is equal to the power received by one port in the antenna with an impedance of 5 × 3 = 15 Ω since for these wavelengths three ports “act” like one. For higher frequencies, the geometry of the meander begins to influence the AFC and to distort it. It is obvious that a decrease in the meander dimensions *L*_1_, *L*_2_, and *L*_3_ leads to a decrease in the described effect. But, in our case, these dimensions were chosen based on the maximum resolution of the standard technology of alignment and magnetron deposition of YBaCuO films on a bicrystal substrate [25]. This problem can be mitigated by using ion irradiation [4,18,26] or step-edge junction technology [19], which will significantly increase the receiving properties and efficiency of the JJ series at high frequencies. While the integral received power of the three-port configuration increases due to better antenna matching, each individual port in the array receives less power than in the case of a single port in the antenna. Due to the serial connection of the JJs into a common receiving system, the elements share the power among them. The more elements there are, the less power goes to each individual port.

Table 1 shows the proportion of absorbed power for each element and the total value depending on the number of junctions in the antenna for a frequency of 250 GHz. Note that the port numbering such that port number 1 is always assigned to the central port and ascending port numbers correspond to ports with increasing distance from the center (see Figure 3). The table shows values for only half of the ports plus the center one because, as discussed earlier, the result for the rest of the ports are identical.

**Table 1:** Absorbed power for different configurations of JJs series integrated into the same log-periodic antenna.

Total number of ports in series	Total absorbed power/incident power	Power absorbed in each port/incident power, *p*_i_					

		*p*_1_	*p*_2_	*p*_3_	*p*_4_	*p*_5_	*p*_6_

1	0.36	0.36					
3	0.65	0.24	0.20				
5	0.81	0.16	0.18	0.14			
7	0.82	0.13	0.11	0.12	0.10		
9	0.75	0.08	0.09	0.08	0.09	0.07	
11	0.70	0.08	0.06	0.07	0.06	0.07	0.05

It can be shown that for the case of a strong mismatch between the impedance of the antenna and the receiving elements, an increase in the series ports number leads to an increase in the total absorbed power as *P*_total_ ≈ *NP*_per_port_, while the power per port *P*_per_port_ remains constant. For the case of matched impedances with an increase in the number of JJs, the total power *P*_total_ remains constant and is divided between all elements such that *P*_per_port_ ≈ *P*_total_/*N*. The situation considered in this work is in the middle between the two cases, that is, the total absorbed power increases with the number of junctions, but *P*_per_port_ decreases. Starting from five JJs in series, *P*_total_ reaches saturation with a level below 1. This is due to the fact that the length of the meander becomes so large that it begins to affect the absorption at a given frequency and cross resonances reduce the receiving properties of the system.

## Numerical Modeling

**Figure 5:**
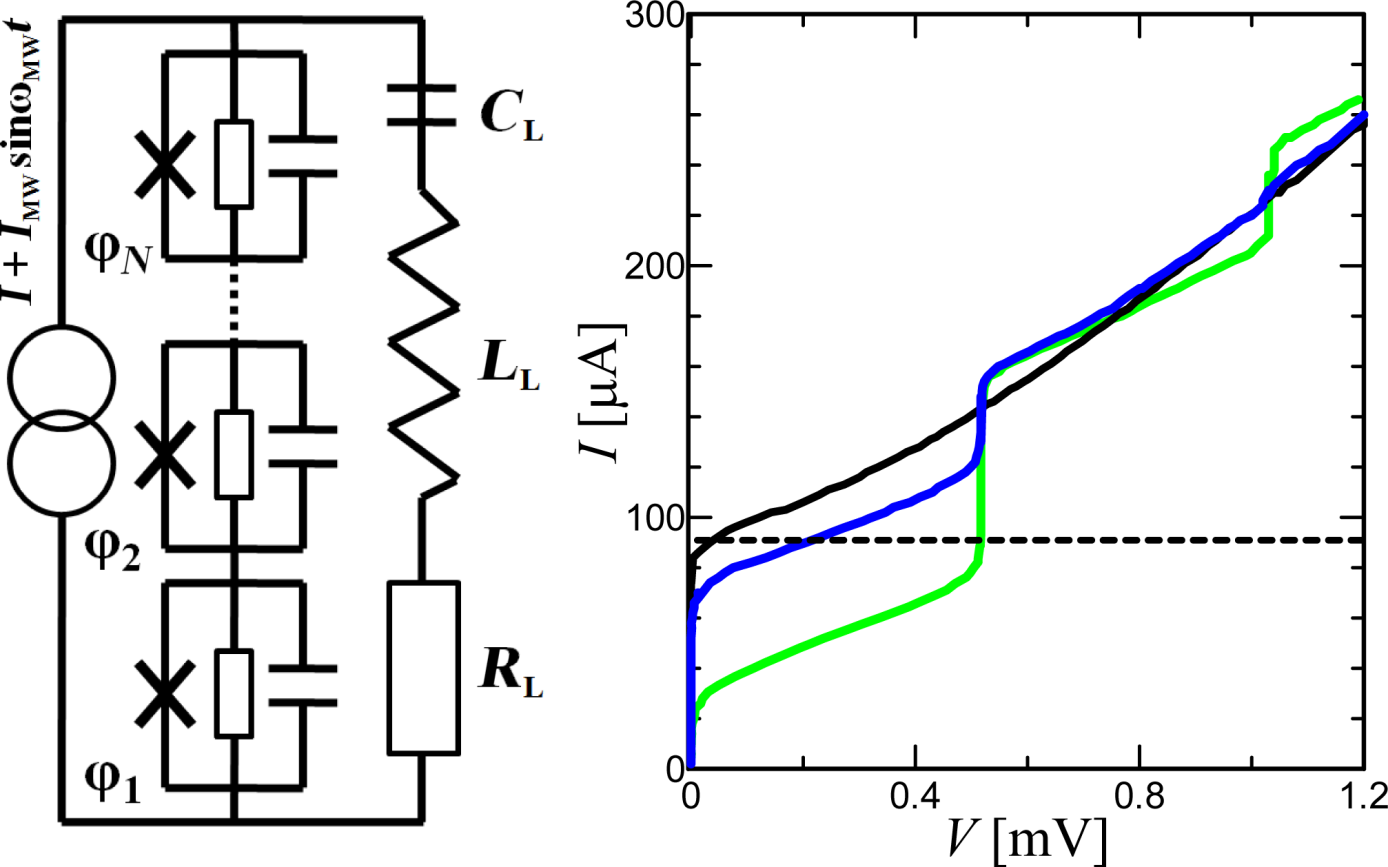
Left: circuit schematic of JJs in a series array interacting via a common RLC load. Right: current–voltage characteristics of a single junction in the absence and under the influence of an external 250 GHz signal, from bottom to top: *P*_MW_ = 250, 50, and 0 nW. The dotted line is the optimal bias current.

Let us now consider a model of an underdamped Josephson junction series array shunted by a load with inductance *L*_L_, resistance *R*_L_, and capacitance *C*_L_ and exposed to an external high-frequency radiation with power *P*_MW_ = *I*_MW_^2^·*R*_N_/2 and frequency *F*_MW_ = ω_MW_/2π. This model consists of a circuit in which there are *N* junctions connected in series one after another with a common load in parallel to all junctions (see Figure 5, left). The circuit equations describing the current–voltage relation are [27–29]:


[2]

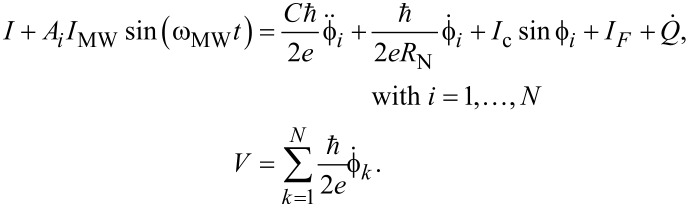



where ϕ*_i_* is the Josephson phase in the *i*-th junction, and the overdot denotes differentiation with respect to time. *I* is the dc current through the junctions, and *V* is the voltage drop across the entire array of series junctions. The amplitude *I*_MW_ of a simple harmonic signal describes the external high-frequency radiation. This signal is multiplied by the coefficient 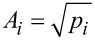, which determines how much of the power received by the antenna is absorbed in a particular JJ (according to Table 1). In Equation 2, the first three terms on the right represent, respectively, the displacement current, the normal current, and the supercurrent with junction critical current *I*_c_, capacitance *C*, and normal resistance *R*_N_ set the same for all JJs in series. Thermal fluctuations *I*_F_ are treated as white Gaussian noise with zero mean and the correlation function


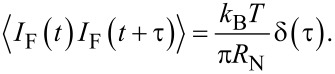


*Q* is the charge on the load capacitor calculated from the equation for the oscillatory circuit:


[3]

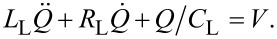



The described model is not complete. To simulate a log-periodic antenna, it is necessary to use an additional system of *K* equations, where *K* is the number of antenna resonances in the frequency range under consideration [30]. Nevertheless, in the described model, the Josephson junctions are connected to each other through the resonant circuit while the electrodynamics is taken into account through the amplitudes of the external signal *A_i_* followed from the previous section. A complete model of the antenna interacting with the nonlinear resonance circuit (Josephson junction) through the Y-matrix is currently being developed. This model will allow one to dynamically calculate the MW current flowing in each of the junctions at a given total high-frequency current in the antenna.

As previously mentioned, the junctions parameters were set the same for all JJs. Although there is always a parameter spread in fabrication, for high temperatures of the order of 70 K this difference is insignificant [3]. Thus, the parameters of the junctions were chosen as typical for bicrystal JJs fabricated by magnetron deposition [24–25,31–33]: *I*_c_ = 100 μA, *R*_N_ = 5 Ω, *C* = 0.02 pF. The antenna parameters were chosen based on the impedance calculation using the relations Re(*Z*) = *R*_L_, dIm(*Z*)/dω = 2*L*_L_, and 
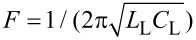
. For the serial resonance at 250 GHz, *R*_L_ = 43 Ω, *L*_L_ = 500 pH, and *C*_L_ = 0.8 fF.

Figure 5, right, shows the current–voltage characteristics (IVCs) of a single junction with the antenna under the influence of an external signal. A bias current regime with the optimum operating point near the critical current (dashed line) is selected for broadband detection. The response is determined by the voltage, which increases with increasing power until it reaches the voltage 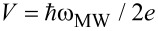 at the Shapiro step. For this regime, the use of serial JJs can be beneficial since the total voltage increases in proportion to the number of junctions.

**Figure 6:**
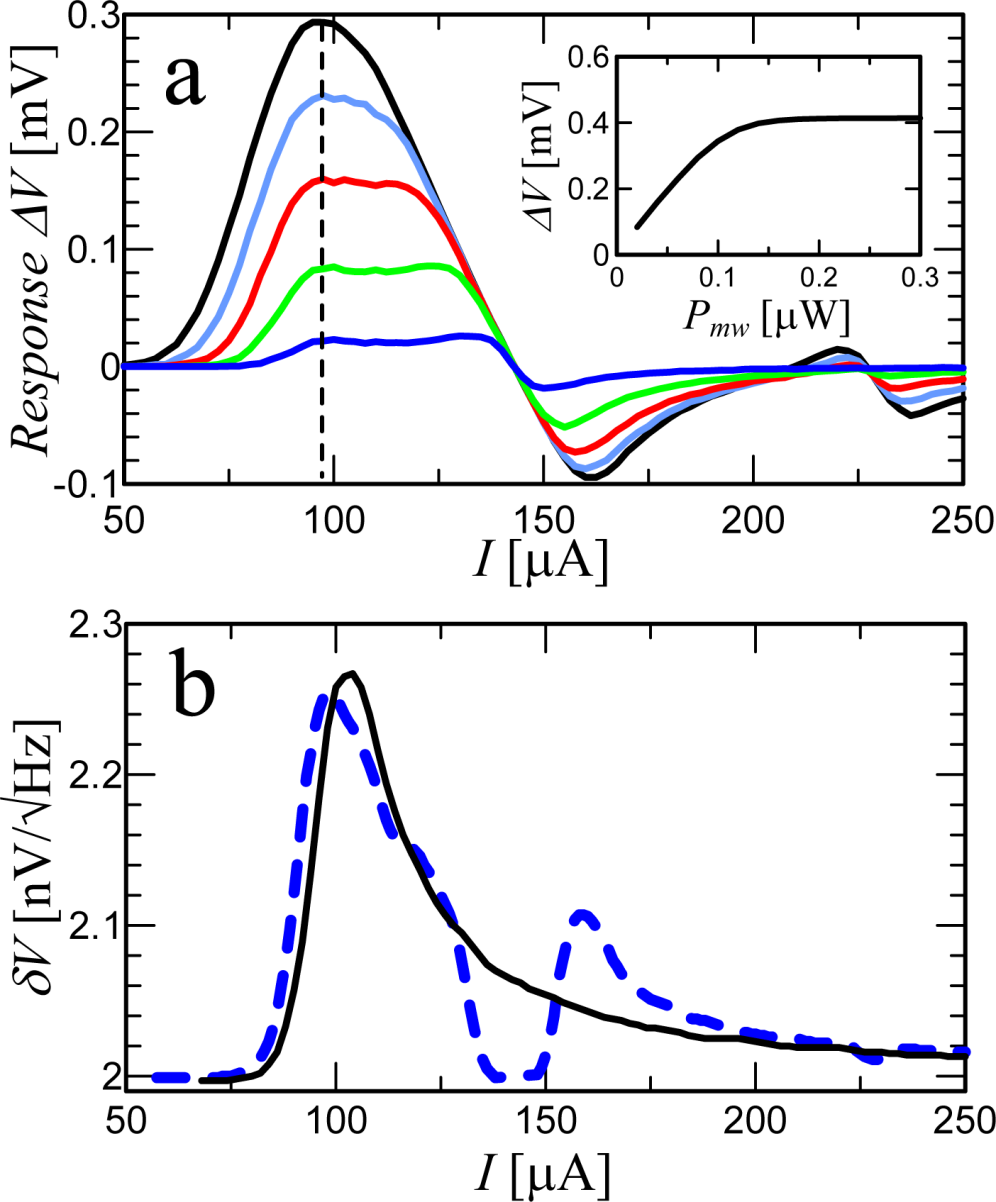
(a) Response Δ*V* versus dc current *I* for different MW signals, from bottom to top: *P*_MW_ = 5, 20, 40, 60, and 80 nW. The dotted line represents the optimal detection regime for the bias current *I* = 98 μA. The inset shows the dependence of the response at the operating point on the magnitude of the external signal. (b) Total output voltage noise of the Josephson junction versus bias current for *P*_MW_ = 0 W (solid curve) and *P*_MW_ = 10 nW (dashed curve).

The first important feature for the characterization of a Josephson junction as a direct detector is the voltage response Δ*V*, that is, the voltage difference between the absence and the presence of an external signal. Figure 6a shows the response as function of the bias current for several power levels *P*_MW_. Qualitatively, the response dependencies coincide with the measured ones in [17]. The dependencies have two maxima, which merge into one with increasing power. The right peak corresponds to the frequency-selective response at a constant voltage of the Shapiro step. The left peak is a classical response with a bias current near the critical one. The inset of Figure 6a shows the Δ*V*(*P*_MW_) dependence at the optimal bias *I* = 98 μA. The response amplitude is a linear function of the radiation power at low values of *P*_MW_ and saturates for *P*_MW_ = 10^−7^ W [17,33–34]. The responsivity *r_V_*, which is a derivative of the Δ*V*(*P*_MW_) dependence, will be discussed later.

The second important parameter is the output voltage noise δ*V*. The theoretical estimation of δ*V* according to the Nyquist formula gives a discrepancy with the experimental values by two orders of magnitude [34–35]. It does not take into account the increase in output noise due to the influence of low-frequency noise spectra of the critical current fluctuations δ*I*_c_ and normal resistance fluctuations δ*R*_N_ associated with the transport mechanisms of the Cooper pairs and quasiparticles, respectively [36–37]. For a Josephson junction having a non-hysteretic IVC, the output voltage spectrum at a fixed bias current is defined as [38]:


[4] $$S_{V}\left(F\right)={\left(V-R_{\text{d}}I\right)}^{2}S_{I}\left(F\right)+V^{2}S_{R}\left(F\right),$$

where *R*_d_ is the differential resistance; 
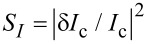
 and 
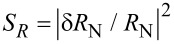
 depend on the nature of the junction barrier. For the considered structures, *S_I_* = 10^−12^ was chosen as a typical value of the current fluctuations at a frequency of 100 Hz [39–40]. For fluctuations of bicrystal YBCO junctions, δ*R*_N_ are mostly insignificant [31,41–42]. In the considered case of the operating point near zero voltage, they do not play a role and are assumed equal to zero *S_R_* = 0. The model also takes into account the amplifier noise, which has a typical value of 
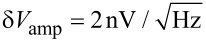
 [43]. Thus, the total output noise for *N* Josephson junctions is


[5] $$\delta V=\sqrt{\delta V_{\text{amp}}{}^{2}+N\cdot S_{V}},$$

and the δ*V*(*I*) dependence is shown in Figure 6b for two power levels.

**Figure 7:**
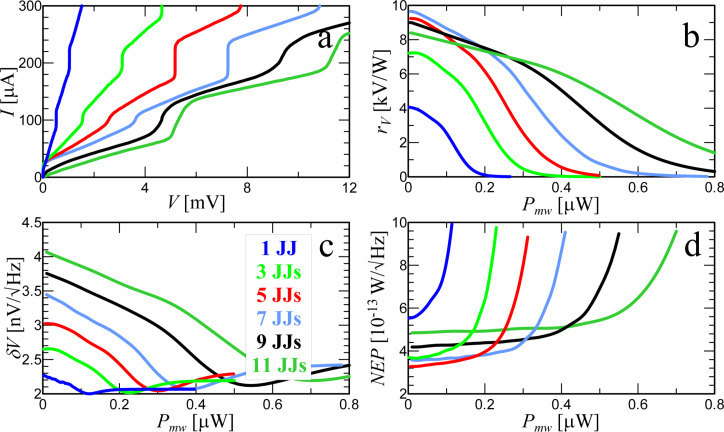
(a) IVCs for different numbers of JJs from one to eleven under a MW signal with power *P*_MW_ = 3 μW and frequency *F*_MW_ = 250 GHz. (b) Responsivity, (c) output voltage noise, and (d) NEP are shown depending on the power of the external signal at an operating point *I* = 98 μA.

Now let us consider the receiving characteristics of structures depending on the number of JJs. Figure 7a shows the current–voltage characteristics for a signal power level *P*_MW_ = 3 μW and different numbers of junctions in series. With an increase in the number of junctions, the Shapiro steps are blurred. This is due to an increase in the spread of the amplitudes *A_i_* in different junctions according to Table 1.

As discussed earlier, the Josephson junction has a constant responsivity for low signal levels. Figure 7b shows the dependence of *r_V_* = d*V*/d*P*_MW_ on the power for the optimal operating current. For a single junction (bottom blue curve) *r_V_* reaches the typical 4000 V/W [34,43–44]. The voltage responsivity can be approximately expressed as [43]


[6] $$r_{V}=R_{d}\cdot A_{1}{}^{2}/\left(2I_{c}R_{N}\Omega^{2}\right),$$

where Ω is the normalized frequency, 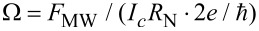. For a single junction with *R_d_* = 12 Ω, *A*_1_^2^ = 0.36 (Table 1), Ω = 1.04 the approximate value *r_V_* = 4025 V/W is close to the numerical one. It is also possible to estimate the response for *N* Josephson junctions according to the additive law:


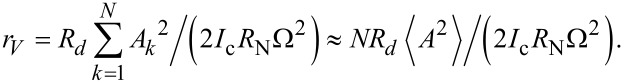


The δ*V*(*P*_MW_) dependence in Figure 7c, which is proportional to 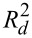 (Equation 4), has a maximum for the highest responsivity and falls to the minimum noise level at the Shapiro step. The receiving system is most comprehensively characterized by the noise-equivalent power


[7] $$\text{NEP}=\delta V/r_{V}.$$

Its value (Figure 7d) for a single JJ of about 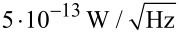 is in agreement with the experimental values [17,34,43–44]. For a configuration of three JJs in series, the responsivity increased by 1.8 times compared to a single junction, and the noise increased by 1.2 times. As a result, the NEP fell by 1.5 times. Note that despite the fact that the responsivity decreases with increasing power, the NEP remains almost unchanged over a wide range of *P*_MW_.

Another important characteristic is the power dynamic range *D* = *P*_S_/*P*_0_. Here *P*_S_ is the upper limit for which the detector response deviates from linearity. The 3 dB criterion is also used as a definition, that is, *P*_S_ is the level of input radiation power, at which the detector responsivity decreases by a factor of two. *P*_0_ is the bottom limit determined by the noise equivalent power and the frequency band Δ*F* of the detection system, 
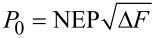
. For a single junction the upper limit of the power dynamic range is *P*_S_ = 110 nW. For eleven JJs in series *P*_S_ = 555 nW. That is, the Josephson junctions in series share the total power among themselves and do not saturate. The power dynamic range *D* increases with an increase in the number of junctions. For 11 JJs, *D* increased by 5.6 times compared to a single junction. This is possible due to the mismatch of the impedances of JJs and antenna. As a result, with an increase in the number of junctions in a chain, the total absorbed power increases and is divided between the JJs.

## Conclusion

The technique for calculating the receiving properties of JJs in the broadband detection regime was developed and applied. At the first stage, electromagnetic modeling of several geometries of antennas was carried out for effective receiving in the frequency range of 50–800 GHz. The electromagnetic properties of the systems were investigated, namely the amplitude–frequency characteristic and the beam pattern. For the chosen antenna geometry, different cases of a series of junctions integrated as a receiving element were considered; the fraction of the absorbed power in each JJ was investigated and it was shown that the use of seven junctions makes it possible to increase the total absorbed power at a fixed frequency of 250 GHz by a factor of 2.3. At the second stage, by solving a system of second-order differential equations related through the equation for the antenna, the characteristics of the detector in the bias current regime were calculated, namely current–voltage characteristic, responsivity, voltage noise, and noise equivalent power. It was shown that the use of a series chain of junctions improves the responsivity by a factor of 2.5, the NEP value by a factor of 1.5, and the dynamic range by a factor of 5. As a result, for the standard technology of YBaCuO magnetron sputtering on a bicrystal substrate for a temperature of 77 K, a NEP in the region of 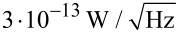 can be obtained.

It is possible to improve the match between the Josephson junction chain and the antenna of the receiving system by reducing the size of the JJs and the meander-type microstrip. This will avoid the observed effect of a decrease in the absorbed power in each junction at high frequencies. In addition, reducing the current noise of JJs can improve NEP and increase the benefit from linking junctions into a series chain. For this it is necessary, first, to perform synchronous detection at frequencies higher than 100 Hz, where the noise falls [45], and second, to reduce the noise by annealing the YBCO bicrystal junction in atomic oxygen [39].
